# Association between geriatric nutritional risk index and fecal incontinence in individuals with stroke: mediating roles of systemic immune inflammation index and oxidative balance scores

**DOI:** 10.3389/fnut.2025.1692314

**Published:** 2025-11-26

**Authors:** Yian Xiang, Zhuang Zhu, Qifang Shi, Kefan Yi

**Affiliations:** 1 Department of Rehabilitation Medicine, Lishui Municipal Central Hospital, The Fifth Affiliated Hospital of Wenzhou Medical University, Lishui, China; 2 Department of Neurology, The First Affiliated Hospital with Nanjing Medical University, Nanjing, China; 3 Department of Intensive Care Medicine, Shanghai Sixth People's Hospital Affiliated to Shanghai Jiao Tong University School of Medicine, Shanghai, China; 4 Department of Clinical Nutrition, Shanghai Deji Hospital, Qingdao University, Shanghai, China

**Keywords:** geriatric nutritional risk index, fecal incontinence, fecal incontinence severity index, systemic immune inflammation index, oxidative balance scores, stroke

## Abstract

**Background:**

This study aims to investigate the association of geriatric nutritional risk index (GNRI) with fecal incontinence (FI) and fecal incontinence severity index (FISI) in individuals with stroke and to explore potential mechanisms underlying this link.

**Methods:**

Data were from the health examination centers of Lishui Municipal Center Hospital and Shanghai Deji Hospital, with 1906 participants. Multivariate logistic regression models, multivariate linear regression models, and restricted cubic spline (RCS) models were applied to assess the association of GNRI with FI and FISI in individuals with stroke. Subgroup analyses and interaction tests were performed to evaluate heterogeneity. Sensitivity analyses were used to test the robustness of the findings. Mediation analysis was employed to investigate the mediating roles of the systemic immune-inflammation index (SII) and oxidative balance score (OBS).

**Results:**

After full adjustment for covariates, GNRI demonstrated a significant linear negative association with both FI (OR = 0.69, 95% CI: 0.53–0.81) and FISI (*β* = −1.08, 95% CI: −1.81 to −0.83) in individuals with stroke. Subgroup analyses confirmed the consistency of these negative associations across all predefined strata, with significant interaction effects observed for BMI categories, smoking status, household income, and diabetes. The SII and OBS were identified as significant mediators of the association of GNRI with FI and FISI in individuals with stroke. Sensitivity analyses revealed that the association of GNRI with both FI and FISI in individuals with stroke remained robust.

**Conclusion:**

GNRI has a significant negative linear association with FI and FISI in individuals with stroke, mediated by the SII index and OBS. These results highlight the critical role of anti-inflammatory interventions and antioxidant strategies in mitigating FI risk in individuals with stroke.

## 1 Introduction

Stroke is an acute cerebrovascular disease caused by damage to brain tissue due to interruption of blood supply to the brain or rupture of blood vessels (1). It is one of the leading causes of disability and death worldwide, causing approximately 6.5 million deaths annually (11% of total global deaths), with approximately three-quarters of these deaths occurring in low- and middle-income countries (2). In addition, some epidemiological data have demonstrated that there are approximately 13.7 million new cases of stroke each year worldwide, and approximately two-thirds of strokes occur in people over the age of 60 (3). Furthermore, some studies show that after the age of 60, the incidence of stroke approximately doubles for every 10-year increase in age (4). Stroke can not only lead to death during the acute phase, but survivors often face a variety of short-term and long-term complications such as motor dysfunction (hemiplegia), language impairment (aphasia), cognitive impairment (dementia), epileptic seizure, deep vein thrombosis, pulmonary embolism, urinary tract infection, and urinary incontinence that severely affect their quality of life and even increase their risk of death (5, 6). Therefore, the high incidence and high disability rate of stroke in the elderly population highlight the importance of public health interventions.

Fecal incontinence (FI), defined as recurrent involuntary bowel movements persisting for ≥1 month in adults, is a common abnormal gastrointestinal symptom (7). Currently, the global prevalence of FI in adults is nearly 8.3%. Especially, the prevalence of FI among people aged 65 and above is approximately 5–10%, while among the elderly aged 85 and above, the rate can be as high as 15–20% (8). In addition, FI is also a common complication of various diseases, such as brain diseases (stroke and dementia), spinal cord diseases (multiple sclerosis and spinal cord tumor), peripheral nerve damage (diabetic peripheral neuropathy), and digestive system diseases (Crohn’s disease, ulcerative colitis, and irritable bowel syndrome) (9–12). Some studies have shown that the underlying causes of FI usually involve damage to the structure or function of the anal sphincter, abnormal nerve control signals, changes in stool consistency, or cognitive impairment (13–15). Since FI is a gastrointestinal tract complication in patients with stroke, existing clinical data have supported that during the acute phase of stroke, the incidence of fecal incontinence can reach up to 40% (16). Currently, the exact risk factors of FI in patients with stroke remain unclear. Some studies have identified several risk factors of FI in patients with stroke, including severe neurological impairment, consciousness disorder, and initial urinary incontinence (17, 18). In addition, recent studies also show that poor nutrition may be an independent risk factor for FI and stroke, as malnutrition increases the risk of muscle atrophy, weakness, and intestinal dysfunction, which aggravates or causes the risk of FI and stroke (19, 20). However, the association between malnutrition and FI in patients with stroke remains unclear.

Malnutrition refers to insufficient, excessive, or imbalanced intake of energy, protein, and/or other nutrients, which has a measurable adverse effect on the body’s composition, function, and clinical outcomes (21). According to data from the World Health Organization (WHO) and the Food and Agriculture Organization (FAO), approximately 735 million people worldwide faced hunger (i.e., chronic food insecurity) in 2022, an increase of 122 million people compared to 2019 (22). Numerous epidemiological studies have demonstrated that malnutrition has profound effects on adult health, significantly increasing the risk of developing various chronic non-communicable disease, including cardiovascular and metabolic diseases (type 2 diabetes, hypertension, and coronary heart disease), musculoskeletal system diseases (osteoporosis, fracture risk, and sarcopenia), and respiratory system diseases (chronic obstructive pulmonary disease) (23–26). This underscores the importance of nutritional risk assessment and nutritional interventions in preventing the onset and progression of chronic diseases. Currently, geriatric nutritional risk index (GNRI), as a malnutrition-related indicator, is widely used to assess nutritional status in elderly people (27). GNRI, calculated from weight, height, and serum albumin levels, has the following advantages compared to MUST and NRS-2002 in assessing nutritional status in elderly people: being highly sensitive to physiological changes in the elderly, being entirely based on objective data to assess nutritional status, and being strongly associated with clinical outcomes, which suggested GNRI effectively and obesitively reflects overall nutritional status (28). Higher GNRI values generally indicate better nutritional health. Recent studies suggest that GNRI may predict gastrointestinal disease and stroke risk (29, 30). For example, a Chinese longitudinal study by Chen et al. demonstrated that GNRI was inversely associated with ulcerative colitis (UC) and Crohn’s disease (CD) risk, with area under the curve (AUC) values of 0.86 and 0.88, respectively, underscoring its value in gastrointestinal disease screening (29). In a multicenter study from China, the authors prospectively collected data on patients with intracerebral hemorrhage, and their findings revealed that low GNRI was significantly associated with poor functional outcomes (mRS score > 2) at 90 days (31). However, the association between GNRI and FI in patients with stroke remains unclear in large population-based studies.

In addition, Inflammation and oxidative stress are essential biological mechanisms that may significantly contribute to the occurrence and development of FI and stroke (32–34). For example, a US cohort study by Young et al. revealed that inflammatory and oxidative stress factors could increase the risk of FI after surgical intervention for inflammatory bowel disease (IBD) patients (35). In addition, in a retrospective study from China, high fibrinogen levels were significantly correlated with increased inflammatory markers such as SII and SIRI among acute ischemic stroke patients (*N* = 1,291) (36). The systemic immune-inflammation (SII) index and oxidative balance scores (OBS), which measure inflammation and oxidative stress levels, are widely used in epidemiological research (37, 38). However, the mediating role of the SII index and OBS in the association between FI in patients with stroke and GNRI remains unclear. Given the critical role of inflammation and oxidative stress in FI progression, we hypothesize that these biomarkers may mediate the association between GNRI and FI in patients with stroke. In this study, we used a health examination center of Lishui Municipal Center Hospital and Shanghai Deji Hospital to explore the association between GNRI and FI in patients with stroke, and the mediation effects of the SII index and OBS.

## 2 Methods

### 2.1 Study design

We designed a retrospective cross-sectional study using population health examination data from January 1, 2022, to January 1, 2024, in the two health examination centers of Lishui Municipal Center Hospital and Shanghai Deji Hospital to explore the association of GNRI and FI in patients with stroke. This retrospective cross-sectional study was approved by the Ethics Committee of Lishui Municipal Center Hospital (LSMCH-2025-16) and Shanghai Deji Hospital (SHDJ-2025-13). Each individual voluntarily signed informed consent documents following the Declaration of Helsinki principles.

**Figure 1:**
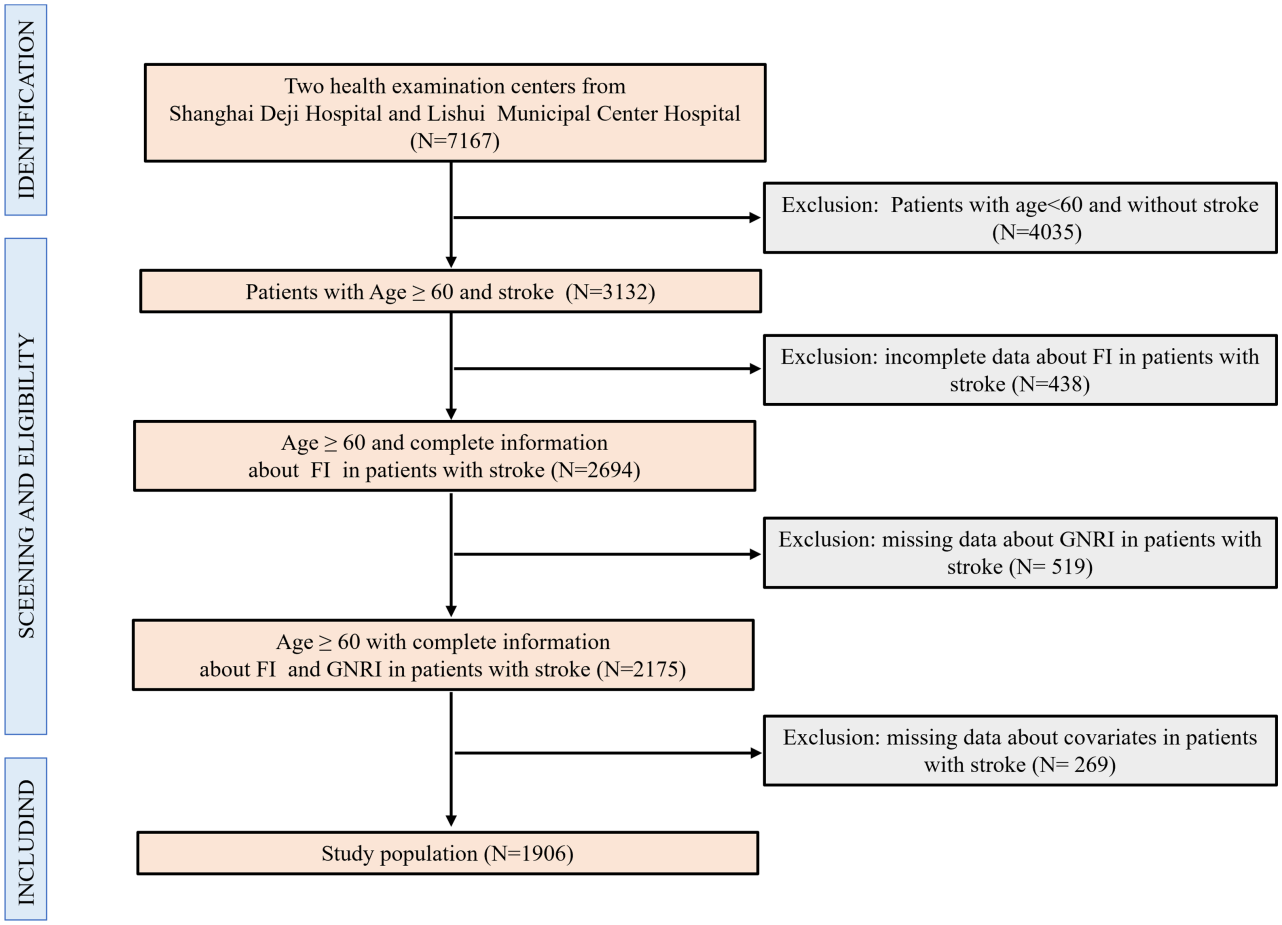
Flow chart of participants in inclusion and exclusion criteria.

In our study, we firstly included 7,167 participants, then excluded participants aged under 60 years and without stroke (*N* = 4,035), those with incomplete data about FI in patients with stroke (*N* = 438), those with missing data about GNRI in patients with stroke (*N* = 519), and those with missing data about covariates in patients with stroke (*N* = 269). Finally, 1906 patients aged 60 years and over with stroke and complete data on FI and GNRI, and covariates were included. The inclusion and exclusion criteria for participants were detailed in Figure 1.

### 2.2 Calculation of GNRI

The calculation formula for GNRI is as follows: GNRI = [41.7 × present weight (kg)/ideal weight (kg)] + [1.489 × serum albumin (g/L)]. The formula for calculating ideal weight is: for males, ideal weight = height (cm) – 100 – [(height – 150)/ 4]; for females, ideal weight = height (cm) – 100 – [(height – 150)/ 2.5] (29). When calculating the GNRI, if the present weight is greater than the ideal weight, the ratio of the present weight to the ideal weight is recorded as 1 (39).

### 2.3 Measurement of SII index and OBS

The SII index was calculated based on the following formula: platelet count (1,000 cells/uL) × neutrophil count (1,000 cells/uL) / lymphocyte count (1,000 cells/uL).

Dietary intake was assessed using a validated 125-item food frequency questionnaire (FFQ) (40). This questionnaire covers all major food categories, including grains, meat, seafood, eggs, dairy, soy products, vegetables (further subdivided into leafy greens, root vegetables, melons and fruits, etc.), fruits, nuts, beverages, and cooking oils (40). The complete food list is detailed in Supplementary Table S1. Participants reported the frequency of consumption and typical portion sizes for each food item over the past year. The nutrient content of each food item (including vitamin C, vitamin E, beta-carotene, total fat, saturated fat, dietary fiber, calcium, magnesium, zinc, total folate, selenium, etc.) was obtained by linking to the Chinese Food Composition Table (Standard Edition) version 2019 (41). The total daily nutrient intake for each participant was calculated by multiplying the consumption of all foods by their respective nutrient values and summing the results.

The OBS was constructed based on 16 dietary components and 4 lifestyle factors to represent an individual’s overall oxidative stress-related exposure (42). Components were classified *a priori* as either pro-oxidant (e.g., saturated fat, iron, smoking) or antioxidant (e.g., vitamin C, vitamin E, carotenoids, physical activity) (42). For each component, intake levels or status were categorized into tertiles, quartiles, or based on clinical guidelines. Antioxidant components were scored from 0 to 2, where higher intakes received higher scores. Conversely, pro-oxidant components were scored from 2 to 0, where higher intakes received lower scores. The scores across all 20 components were summed to yield the overall OBS, with a higher total score indicating a greater preponderance of antioxidant exposures (43). The complete list of components, along with their specific cutoff values and assigned scores, is detailed in Supplementary Table S2.

### 2.4 Definition of stroke, FI, and FISI

National Institutes of Health Stroke Scale (NIHSS), core clinical criteria (based on medical history and symptoms), and neuroimaging examination were used to diagnose stroke. The Bowel Health Questionnaire (BHQ) was used to obtain information related to FI and the intestinal function of study participants. In the BHQ, participants used the Bristol Stool Form Scale (BSFS) cards to assess their most common stool type and answered questions about the type and frequency of accidental bowel leakage experienced in the past 30 days (44). FI was defined as the participants with involuntary discharge of mucus, liquid, or solid stools in the past 30 days (45). The fecal incontinence severity index (FISI), a quantitative measure of fecal incontinence severity, was assessed using the FISI questionnaire (46). It assessed four types of incontinence (gas, mucus, liquid, and solid stool) across six frequency levels, ranging from never to two or more occurrences per day. Each frequency level for the four types of incontinence was assigned a weighted score ranging from 0 to 19, and the FISI was calculated as the sum of these scores (47). The detailed scoring for the four types of incontinence was provided in Supplementary Table S3. The higher scores on the FISI indicated more severe fecal incontinence (48).

### 2.5 Assessment of covariates

Our study included the following sociodemographic, lifestyle, and clinical biomarker covariates. Sociodemographic and lifestyle covariates included age, gender (female and male), education levels (less than high school, high school, more than college), and poverty index ratio (PIR) [<1.0 (below the poverty threshold), 1.0–3.0 (low to middle income), and ≥ 3.0 (middle to high income)], body mass index (BMI) [<18.5 (underweight), 18.5–24.9 (normal weight), 25–29.9 (overweight), ≥30 kg/m^2^ (obese)], smoking status (never, former, current) and drinking status (never, former, current). Hypertension was defined as participants with an average of three times systolic blood pressure (SBP) ≥ 130 and diastolic blood pressure (DBP) ≥ 80 mmHg, or diagnosed by a physician, or usage of lower blood pressure medicine, which was categorized into yes or no. Diabetes was defined as participants with fasting glucose ≥ 126 mg/dL or HbA1c ≥ 6.5%, or diagnosis by a physician, or usage of lower blood glucose medicine, which was categorized into yes or no (49, 50). Chronic kidney disease (CKD) was defined as apparent signs of kidney damage lasting more than 3 months, including albuminuria, abnormal urine sediment, tubular-related lesions, abnormal renal histopathology, structural abnormalities visible on renal imaging, and decreased glomerular filtration rate (GFR) (GFR < 60 mL/min/1.73 m^2^), which was categorized as yes or no. Chronic obstructive pulmonary disease (COPD) was defined as an individual with persistent respiratory symptoms (chronic cough, chronic cough with phlegm, dyspnea, and wheeze) and a pulmonary function test (after inhaling a bronchodilator, the ratio of forced expiratory volume in one second (FEV₁) to forced vital capacity (FVC) is < 0.70), which was categorized into yes or no. Clinical biomarkers covariates included waist circumference (WC), total cholesterol (TC), triglyceride (TG), low-density lipoprotein cholesterol (LDL), high-density lipoprotein cholesterol (HDL), glycated hemoglobin A1c (HbA1c), fast glucose, C-reactive protein (CRP), uric acid (UA), total cholesterol (TC), albumin (ALB), aspartate aminotransferase (AST), alanine aminotransferase (ALT), gamma-glutamyl transferase (GGT), lactate dehydrogenase (LDH), and Interleukin-6 (IL-6), and Interleukin-12 (IL-12).

### 2.6 Statistical analysis

In this study, the baseline characteristics data of participants were statistically analyzed using a *t*-test (continuous variable) and a chi-square test (categorical variable). GNRI was analyzed as both a continuous variable and a categorical variable, divided into quartiles: Q1 (lowest), Q2, Q3, and Q4 (highest). Multivariate logistic and linear regression analyses were used to assess the association between GNRI, FI, and FISI in individuals with stroke. We also used restricted cubic splines (RCS) models to assess the non-linear relationship of GNRI with FI and FISI in individuals with stroke. Subgroup analyses were performed to evaluate the stability of the association between GNRI, FI, and FISI in individuals with stroke across subgroups stratified by age, gender, education level, PIR, BMI, drinking status, smoking status, physical activity levels, hypertension, diabetes, COPD, and CKD. The discriminative capacity of GNRI was assessed by net reclassification improvement (NRI) and integrated discrimination improvement (IDI). Several sensitivity analyses were conducted to examine the robustness of the association between GNRI, FI, and FISI in individuals with stroke.

The mediation analysis was used to explore the mediation effect of SII and OBS in the association between GNRI and FI and FISI. In addition, we explore the interaction effect of OBS and SII in the mediation analysis. All statistical analyses were performed using R (version 4.3.0). Statistical significance was set at *p* < 0.05 with a two-sided test.

## 3 Results

### 3.1 Baseline characteristics of the participants

The baseline characteristics of the participants are presented in Table 1. Among the 1906 participants who met the inclusion and exclusion criteria, of whom 52.15% (*N* = 994) were male, 46.48% (*N* = 886) had a college degree or above educational level, and 24.03% (*N* = 458) had FI. Significant differences were observed in all covariates between FI and non-FI groups in participants with stroke (all *p* < 0.05).

**Table 1:** Baseline characteristics of the participants with stroke.

Variables	Total (*n* = 1906), *n* (%)	FI (*n* = 458), *n* (%)	Non-FI (*n* = 1,448), *n* (%)	*P*-value
Age	70.67 (8.02)	73.20 (8.71)	68.34 (7.21)	<0.001
Gender				<0.001
Female	912 (47.85)	207 (45.20)	705 (48.68)	
Male	994 (52.15)	251 (54.80)	743 (51.32)	
Educational levels				0.041
Less than High-school	426 (22.35)	181 (39.51)	245 (16.91)	
High school	594 (31.16)	121 (26.42)	473 (32.66)	
College or above	886 (46.48)	156 (34.06)	730 (50.43)	
Poverty index ratio (PIR)				<0.001
PIR < 1	301 (15.79)	176 (38.42)	125 (8.63)	
1 ≤ PIR < 3	449 (23.55)	150 (32.75)	299 (20.65)	
PIR ≥ 3	1,156 (60.66)	132 (28.83)	1,024 (70.72)	
BMI				<0.001
Underweight	74 (3.88)	28 (6.12)	46 (3.17)	
Normal weight	914 (47.95)	131 (28.60)	783 (54.07)	
Overweight	694 (36.42)	191 (41.70)	503 (34.73)	
Obesity	224 (11.75)	108 (23.58)	116 (8.03)	
Drinking status				<0.001
Yes	635 (33.31)	172 (37.55)	463 (31.97)	
No	1,271 (66.69)	286 (62.45)	985 (68.03)	
Smoking status				<0.001
Never	153 (8.03)	36 (7.86)	117 (8.08)	
Former	679 (35.62)	137 (29.91)	542 (37.44)	
Current	1,074 (56.35)	285 (62.23)	789 (54.48)	
Physical activity				<0.001
Vigorous level	284 (14.90)	102 (22.27)	182 (12.56)	
Middle level	815 (42.76)	153 (33.41)	662 (45.72)	
Other levels	807 (42.34)	203 (44.32)	604 (41.72)	
Hypertension				<0.001
Yes	612 (32.11)	217 (47.38)	395 (27.28)	
No	1,294 (67.89)	241 (52.62)	1,053 (72.72)	
Diabetes				
Yes	594 (31.16)	117 (25.55)	477 (32.94)	<0.001
No	1,312 (68.84)	341 (74.45)	971 (67.06)	
COPD				<0.001
Yes	478 (25.08)	187 (40.83)	291(20.10)	
No	1,428 (74.92)	271 (59.17)	1,157 (79.90)	
CKD				<0.001
Yes	429 (22.51)	168 (36.68)	261 (18.02)	
No	1,477 (77.49)	290 (63.32)	1,187 (81.98)	
GNRI	86.44 (13.63)	81.62 (14.82)	90.29 (12.6)	<0.001
FISI	3.69 (2.19)	16.8 (3.69)	2.43 (1.42)	<0.001
TC (mg/dL)	222.14 (6.35)	228.35 (6.89)	218.45 (5.39)	<0.001
TG (mg/dL)	128.28 (5.58)	133.46 (6.38)	125.39 (4.67)	<0.001
HDL (mg/dL)	39.22 (4.36)	37.17 (5.19)	42.05 (3.62)	<0.001
HbA1c (%)	5.69 (1.16)	6.58 (1.48)	4.68 (1.04)	<0.001
Fast glucose (mg/dL)	94.04 (6.24)	96.27 (7.16)	91.67 (5.28)	<0.001
CRP (mg/L)	3.28 (0.91)	3.95 (1.43)	2.79 (0.70)	<0.001
UA (mg/dL)	5.93 (1.66)	6.72 (2.23)	5.05 (1.24)	<0.001
AST (U/L)	22.16 (3.26)	24.18 (3.69)	20.72 (2.95)	<0.001
ALB (g/L)	46.02 (3.57)	49.05 (3.91)	44.24 (3.16)	<0.001
ALT (U/L)	25.04 (4.25)	27.16 (5.36)	22.24 (3.27)	<0.001
GGT (U/L)	37.06 (3.46)	39.31 (4.26)	34.19 (3.09)	<0.001
LDH (U/L)	141.18 (6.35)	144.67 (7.66)	138.06 (5.48)	<0.001
IL-6 (pg/mL)	2.67 (0.37)	3.14 (0.65)	2.43 (0.23)	<0.001
IL-12 (pg/mL)	2.36 (0.26)	2.79 (0.47)	2.14 (0.18)	<0.001

PIR: family income-to-poverty ratio, BMI: body mass index, WC: waist circumference, COPD: Chronic obstructive pulmonary disease, CKD: Chronic kidney disease, TC: total cholesterol, TG: triglyceride, LDL: low-density lipoprotein cholesterol, HDL: high-density lipoprotein cholesterol, HbA1c: glycated hemoglobin A1c, CRP: C-reactive protein, UA: uric acid, ALB: albumin, ALT: alanine aminotransferase, AST: aspartate aminotransferase, GGT: gamma-glutamyl transferase, LDH: lactate dehydrogenase, ASCVD: atherosclerotic cardiovascular disease. Categorical variables were presented as %, with χ^2^ tests comparing statistical differences between groups. Continuous variables were reported as mean ± standard deviation (SD), with Student’s *t*-test in between-group comparisons. *P* < 0.05 was regarded as statistically significant.

### 3.2 Association between GNRI, FI, and FISI in individuals with stroke

**Figure 2:**
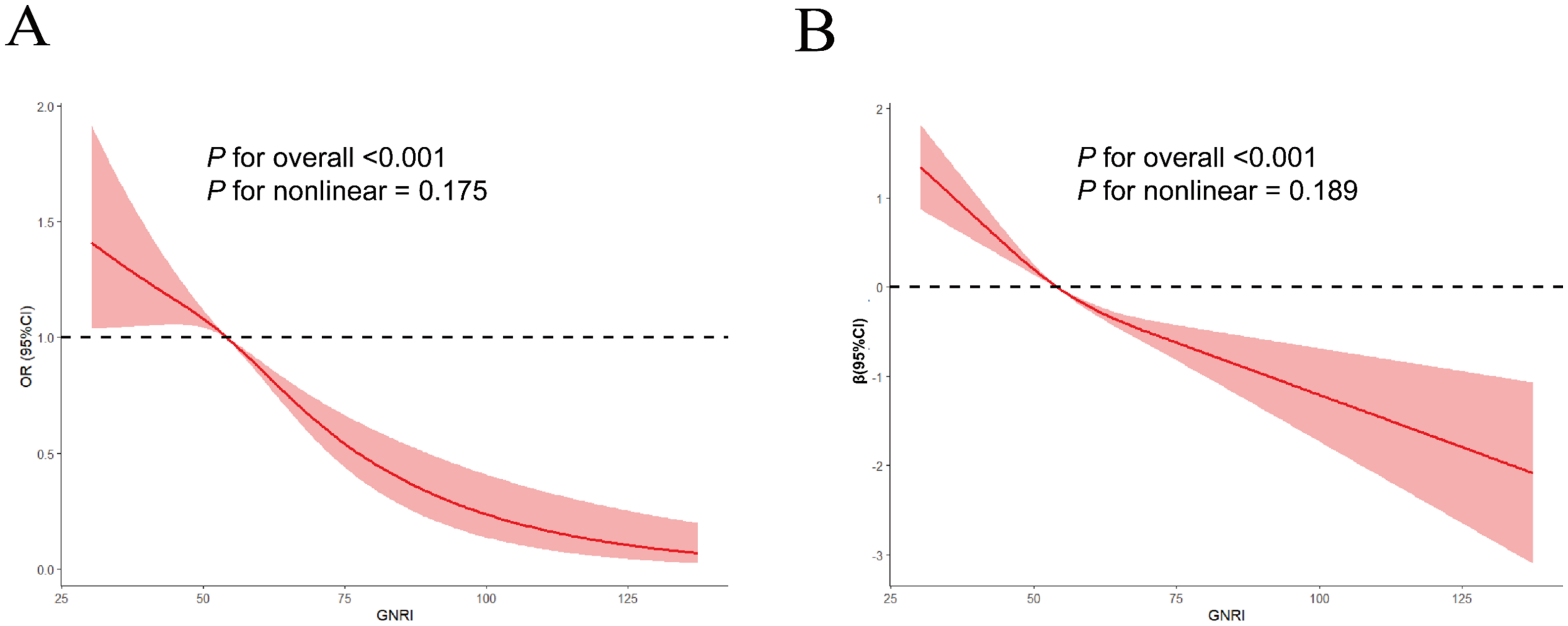
The linear relationship of GNRI with FI **(A)** and FISI **(B)** in RCS models after adjusting all covariates. The solid curve represents the estimated odds ratio (OR) of FI and β of FISI, and the shaded area represents the 95% confidence interval, derived from a restricted cubic spline model with 4 knots. The figure demonstrates a linear decrease in FI odds and FISI with increasing GNRI.

Table 2 showed a significant negative association between GNRI and both FI and FISI in individuals with stroke. For each unit increase in GNRI, the odds ratio of FI in individuals with stroke decreased by 33% in Model I (OR = 0.67, 95% CI: 0.55–0.82), 36% in Model II (OR = 0.64, 95% CI: 0.56–0.78), and 31% in Model III (OR = 0.69, 95% CI: 0.53–0.81). When GNRI was categorized into quartiles, the Q4 group (vs. Q1) showed a 43% reduction for odds ratio of FI in individuals with stroke in Model I (OR = 0.57, 95% CI: 0.35–0.72), 49% in Model II (OR = 0.51, 95% CI: 0.38–0.67), and 46% in Model III (OR = 0.54, 95% CI: 0.41–0.69). Similarly, each unit increase in GNRI was associated with a reduction for FISI scores of individuals with stroke by 1.16 (Model I: *β* = −1.16, 95% CI: −1.80 to −0.70), 1.24 (Model II: *β* = −1.24, 95% CI: −1.82 to −0.86), and 1.08 (Model III: *β* = −1.08, 95% CI: −1.81 to −0.83). Compared to Q1, the Q4 group had a 1.65-point decrease in FISI of individuals with stroke in Model I (*β* = −1.65, 95% CI: −2.55 to −0.89), 1.51 in Model II (*β* = −1.51, 95% CI: −2.41 to −1.16), and 1.43 in Model III (*β* = −1.43, 95% CI: −2.25 to −1.08). Trend analysis revealed a significant linear relationship between GNRI, FI, and FISI in individuals with stroke (all *p* < 0.001). RCS curves further confirmed linear associations for GNRI with FI (P for nonlinear = 0.175, P for overall < 0.001) and FISI (*P* for nonlinear = 0.189, *P* for overall < 0.001) in individuals with stroke (Figures 2A,B).

**Table 2:** Association of GNRI with FI and FISI in individuals with stroke.

Models	OR (95%CI)	*P*-value	β (95%CI)	*P*-value
Model I				
Continuous	0.67 (0.55, 0.82)	<0.001	−1.16 (−1.80, −0.70)	<0.001
Q1	*Ref* (1)		*Ref* (0)	
Q2	0.90 (0.76, 1.13)	0.803	−0.28 (−0.82, 0.30)	0.243
Q3	0.81 (0.67, 0.96)	0.027	−0.55 (−1.16, −0.08)	0.023
Q4	0.57 (0.35, 0.72)	<0.001	−1.65 (−2.55, −0.89)	<0.001
*P* for trend	<0.001		<0.001	
Model II				
Continuous	0.64 (0.56, 0.78)	<0.001	−1.24 (−1.78, −0.79)	< 0.001
Q1	*Ref* (1)		*Ref* (0)	
Q2	0.93 (0.79, 1.12)	0.479	−0.16 (−0.76, 0.33)	0.313
Q3	0.79 (0.61, 0.94)	0.039	−0.76 (−1.21, −0.14)	0.001
Q4	0.51 (0.38, 0.67)	<0.001	−1.51 (−2.41, −1.16)	<0.001
*P* for trend	<0.001		<0.001	
Model III				
Continuous	0.69 (0.53, 0.81)	<0.001	−1.08 (−1.83, −0.73)	<0.001
Q1	*Ref* (1)		*Ref* (0)	
Q2	0.96 (0.80, 1.09)	0.427	−0.11 (−0.70, 0.29)	0.289
Q3	0.82 (0.68, 0.97)	0.046	−0.62 (−1.07, −0.10)	0.005
Q4	0.54 (0.41, 0.69)	<0.001	−1.43 (−2.25, −1.08)	<0.001
*P* for trend	<0.001		<0.001	

Q1: quartile 1; Q2: quartile 2; Q3: quartile 3; Q4: quartile 4. Ref: reference. OR: odds ratio. Model I is a crude model. Model II adjusted for age, gender, educational levels, and PIR. Model III adjusted for all covariates. P < 0.05 was regarded as statistically significant.

### 3.3 Subgroup analyses

In this study, subgroup analyses were conducted to assess the robustness of the association between GNRI and both FI and FISI in individuals with stroke across strata of gender, education level, PIR, BMI, drinking status, smoking status, physical activity, hypertension, diabetes, COPD, and CKD. As shown in Table 3 and Supplementary Table S4, GNRI was negatively associated with FI and FISI in individuals with stroke stratified by specific subgroups (e.g., participants aged <60 years: OR = 0.69, 95% CI: 0.59–0.84; *β* = −1.14, 95% CI: −1.65, −0.73). Additionally, BMI, smoking status, household income, and diabetes significantly modified these associations (*P* for interaction < 0.05).

**Table 3:** Association between GNRI and FI in individuals with stroke stratified by various subgroups.

Subgroups	OR (95% CI)	*P*-value	*P* for interaction
Gender			0.614
Female	0.63 (0.55, 0.87)	<0.001	
Male	0.79 (0.69, 0.91)	0.001	
Educational levels			0.192
Less than High-school	0.58 (0.46, 0.75)	<0.001	
High school	0.63 (0.56, 0.74)	<0.001	
College or above	0.73 (0.61, 0.85)	<0.001	
Poverty index ratio			0.027
PIR < 1	0.62 (0.49, 0.77)	<0.001	
1 ≤ PIR < 3	0.94 (0.78, 1.24)	0.192	
PIR ≥ 3	0.65 (0.51, 0.84)	<0.001	
BMI			0.014
Underweight	0.43 (0.32, 0.65)	<0.001	
Normal weight	0.96 (0.84, 1.32)	0.271	
Overweight	0.59 (0.43, 0.82)	<0.001	
Obesity	0.56 (0.28, 0.97)	0.001	
Drinking status			0.461
Yes	0.75 (0.49, 1.06)	0.072	
No	0.95 (0.76, 1.24)	0.312	
Smoking status			0.001
Never	0.94 (0.73, 1.21)	0.383	
Former	0.65 (0.51, 0.93)	0.006	
Current	0.43 (0.15, 0.68)	<0.001	
Physical activity			0.763
Vigorous level	0.89 (0.68, 0.98)	0.038	
Middle level	0.63 (0.47, 0.91)	0.008	
Other levels	0.54 (0.38, 0.76)	<0.001	
Hypertension			0.952
Yes	0.47 (0.28, 0.72)	<0.001	
No	0.69 (0.51, 0.92)	0.005	
Diabetes			0.004
Yes	0.56 (0.39, 0.84)	<0.001	
No	0.89 (0.77, 1.18)	0.261	
COPD			0.065
Yes	0.84 (0.57, 1.08)	0.089	
No	0.68 (0.53, 0.84)	<0.001	
CKD			0.579
Yes	0.78 (0.65, 0.92)	< 0.001	
No	0.71 (0.59, 0.87)	< 0.001	

PIR: poverty index ratio, BMI: body mass index, COPD: chronic obstructive pulmonary disease, CKD: chronic kidney disease, OR: odds ratio. All covariates were adjusted in the model. *P* < 0.05 was regarded as statistically significant.

### 3.4 Increased predictive effect of GNRI on the FI in individuals with stroke

In this study, we employed IDI and NRI to assess the predictive capacity of GNRI on the FI of the ASCVD. As shown in Table 4, GNRI increases discrimination capacity for the diagnosis of FI in individuals with stroke with NRI (0.135, 95%CI: 0.093–0.186, *p*-value < 0.001) and IDI (0.041, 95%CI: 0.025–0.069, *p*-value < 0.001), which suggests GNRI had a superior predictive performance for the diagnosis of FI in individuals with stroke.

**Table 4:** Discrimination performance of the diagnosis of FI in individuals with stroke.

Models	NRI (95%CI)	*P*-value	IDI (95%CI)	*P*-value
Basic model	Reference		Reference	
Basic model +GNRI	0.135 (0.093, 0.186)	<0.001	0.041 (0.025, 0.069)	<0.001

NRI: net reclassification improvement, IDI: integrated discrimination improvement. Basic model included all covariates. *P* < 0.05 was regarded as statistically significant.

### 3.5 The mediation effects of the SII index and OBS

**Figure 3:**
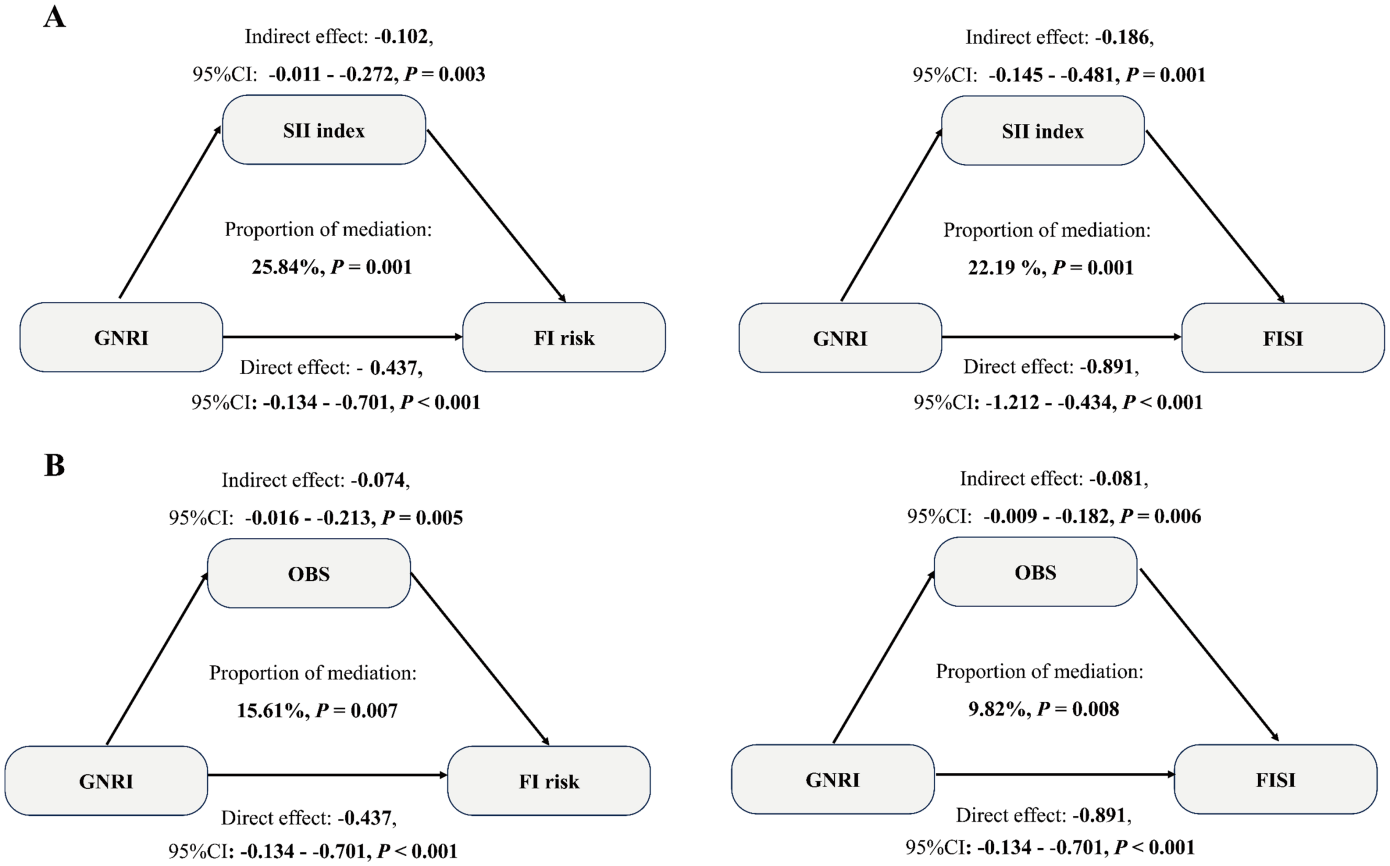
Mediation effects of SII index **(A)** and OBS **(B)** in the association between GNRI and FI and FISI in individuals with stroke. Solid lines represent significant paths. The indirect effect of the SII index and OBS, and the direct effect of GNRI, were calculated. The model reveals that both SII and OBS are significant mediators, explaining a substantial proportion of the total effect.

A mediation analysis was conducted to explore the effects of the SII index and OBS. Supplementary Table S5 showed the association of the SII index and OBS with FI and FISI in individuals with stroke. After adjusting for all covariates, the SII index was positively associated with FI (OR = 1.45, 95% CI: 1.13–1.89) and FISI (*β* = 0.72, 95% CI: 0.34–1.36), while OBS was negatively associated with FI (OR = 0.73, 95% CI: 0.54–0.91) and FISI (β = −0.86, 95% CI: −1.48 – –0.21) (Supplementary Table S5). Supplementary Table S6 revealed the association of the SII index and OBS with GNRI in individuals with stroke. As shown in Supplementary Table S6, after adjustment for all covariates, the SII index showed a negative relationship with GNRI (*β* = −0.56, 95% CI: −0.98 to −0.23), whereas OBS demonstrated a positive relationship (*β* = 0.78, 95% CI: 0.23–1.64). Furthermore, Figures 3A,B indicated that the SII index and OBS mediated 25.84 and 22.19% (SII), and 15.61 and 9.82% (OBS) of the associations of GNRI with FI and FISI in individuals with stroke, respectively. In addition, we further explore the potential interaction effect of OBS and SII on this association. Our results also revealed that SII and OBS have a significant interaction effect on this association of GNRI with FI (OR = 1.24, 95%CI: 1.06–1.59) and FISI (*β* = 0.16, 95%CI: 0.02–0.45), and the simple slopes analysis results demonstrated that the detrimental effect of high SII on FI was most pronounced among individuals with a low OBS. Conversely, this effect was substantially attenuated among those with a high OBS (Supplementary Figures S1, S2).

### 3.6 Sensitivity analyses

In this study, we conducted several sensitivity analyses to examine the stability of the association between GNRI, FI, and FISI in individuals with stroke. To minimize the impact of excessive missing data on the associations among GNRI, FI, and FISI in individuals with stroke, multiple imputation methods were applied, and Supplementary Table S7 showed that the significant negative association of GNRI with FI and FISI in individuals with stroke remained robust after imputation missing data. Additionally, to minimize the effect of comorbidity on the association between GNRI, FI, and FISI in individuals with stroke, we explore the association between GNRI, FI, and FISI after deleting the individuals with hypertension, diabetes, COPD, and CKD. Our results showed that the negative association of GNRI with FI and FISI in individuals with stroke remained consistent in Supplementary Table S8.

## 4 Discussion

Stroke is one of the leading causes of disability and death worldwide and has serious health risks and complications (51). Some evidence showed that approximately 75% of survivors of stroke may experience varying degrees of complications in various organs and tissues, including nervous system diseases (hemiplegia, aphasia, epilepsy, and dysphagia), post-stroke pneumonia, post-stroke depression, incontinence or constipation, urinary retention, etc. (52–56). Although the exact causes of complications in patients with stroke remain unclear, currently, some studies have demonstrated some risk factors (family genetic history, elderly, comorbidities, postoperative activity level, and infection) that increase the risk of these complications for stroke (57, 58). Recent studies also showed that the nutritional status of an individual may affect the risk of complications of stroke. Malnutrition increases the risk of gastrointestinal disease in patients with stroke in a large cohort (59). The association of FI, as a complication of stroke, with the nutritional status of patients with stroke and the potential mechanism remains unclear. Therefore, in this study, we designed a retrospective cross-sectional study to explore the association between GNRI and both FI and FISI in individuals with stroke using data from the two health examination centers of Lishui Municipal Center Hospital and Shanghai Deji Hospital.

In our study, GNRI showed a significant negative linear association with FI and FISI in individuals with stroke after adjusting for relevant covariates. The negative associations remained consistent across all subgroups. This finding positions GNRI not merely as a marker of general nutritional status but as a potential key indicator of anorectal and pelvic floor health. The negative relationship suggests that individuals with poorer nutritional status (lower GNRI) are at a significantly elevated risk for FI. This can be mechanistically explained by the role of malnutrition in promoting sarcopenia, which can affect the striated muscles of the pelvic floor and the external anal sphincter, compromising their contractile strength and structural support (60, 61). Furthermore, protein-energy malnutrition and micronutrient deficiencies can impair peripheral nerve function, potentially leading to reduced sensory perception in the rectum and diminished anal sphincter control, which are critical for maintaining continence (62). Therefore, our results highlight the importance of nutrition support to patients with stroke in lowering the risk of their complications.

It is noteworthy that our results of subgroup analysis revealed that BMI, smoking status, household income, and diabetes significantly modified the associations among GNRI, FI, and FISI in individuals with stroke. Although the exact mechanisms remain unclear, evidence suggests that these associations vary across weight categories, smoking status, income levels, and diabetes. For example, stroke patients with abnormal weight are more prone to gastrointestinal dysfunction (e.g., diarrhea), which accelerates intestinal motility and leads to loose stools that challenge anal sphincter control, thereby increasing FI risk (63). Additionally, abnormal weight may disrupt neural regulation of the anal sphincter in patients with stroke, further elevating FI susceptibility (64, 65). Smoking may dysregulate inflammation-related genes (e.g., *TSG6*, involved in intestinal muscle repair) and barrier function proteins (e.g., *REG3G*) in patients with stroke, impairing anal sphincter synthesis of patients with stroke and increasing FI risk (66–69). Smoking is also linked to impaired gastrointestinal absorption of vitamin D, calcium, and magnesium, potentially compromising anal sphincter repair in patients with stroke, increasing FI risk (70, 71). Low household income and diabetes are associated with chronic inflammation and oxidative stress, exacerbating malnutrition and FI risk in patients with stroke (72, 73). Future longitudinal and animal studies are needed to validate these associations and elucidate their underlying mechanisms.

Inflammation and oxidative stress are critical drivers of fecal incontinence (FI) and stroke onset and progression. A prospective cohort study of 57,432 participants from the Nurses’ Health Study demonstrated that pro-inflammatory diets increase FI risk in older women (74). A national cohort of middle-aged and older Chinese adults showed that participants with elevated levels of both AIP and hs-CRP had the highest risks of stroke (HR: 2.207; 95% CI: 1.771–2.749) (75). For the relationship of oxidative stress factor with FI and stroke, some studies have revealed that oxidative stress-induced free radicals may impair anal sphincter function, contributing to FI (76). In addition, a prospective cohort study revealed that patients with severe stroke were associated with significantly higher malondialdehyde (MDA) levels, as an oxidative stress biomarker, compared to moderate and mild cases (77). Therefore, we hypothesize that inflammation and increasing oxidative stress mediate the association between GNRI, FI, and FISI in patients with stroke. Although the SII index (a systemic inflammation marker) and OBS (an oxidative stress score) are widely used in population studies, their mediating roles in the association between GNRI, FI, and FISI in patients with stroke remain unexplored. In this study, mediation analysis revealed that SII and OBS significantly mediated these associations, with mediation proportions of 25.84% (SII for FI), 22.19% (SII for FISI), 15.61% (OBS for FI), and 9.82% (OBS for FISI). The findings suggest a potential framework for targeted interventions in clinical practice. Firstly, the strong direct effect of GNRI underscores the importance of overall nutritional support as a foundational strategy in decreasing the risk of FI. Secondly, the significant mediating roles of SII and OBS point to the value of adding anti-oxidative and anti-inflammatory therapies. For instance, in individuals identified with high SII, anti-inflammatory interventions (such as the use of specific anti-inflammatory nutrients like omega-3 fatty acids or certain pharmacological agents) could be prioritized. Concurrently, for those with a low OBS, nutritional counseling focused on increasing the intake of antioxidant-rich foods (e.g., fruits, vegetables, nuts) and promoting physical activity could be implemented to improve their oxidative balance.

In addition, our findings of mediation analysis suggest that systemic inflammation and oxidative stress may be a significant pathway influencing the association between GNRI and FI in patients with stroke, which can be mechanistically explained by the detrimental effects of pro-inflammatory cytokines on neuromuscular structures. Specifically, elevated levels of cytokines such as TNF-*α* and IL-6 have been shown to directly impair the function and survival of enteric neurons, leading to disrupted gut motility and visceral hypersensitivity (78, 79). Furthermore, chronic inflammation can activate enteric glial cells, which in turn exacerbate neuronal dysfunction and contribute to the pathophysiology of gut disorders (80). Beyond the enteric nervous system, systemic inflammation is a known driver of sarcopenia. It is plausible that this pro-catabolic state also affects the striated muscles of the pelvic floor, compromising their strength and structural support, which is crucial for normal function (81). Conversely, a higher OBS, reflecting a preponderance of antioxidant exposures, appears to exert a protective effect, potentially through mitigating the pathways described above. The antioxidant components in the OBS, such as vitamins C and E, and various flavonoids, can neutralize reactive oxygen species (ROS) in the gastrointestinal tract, thereby shielding enteric neurons and smooth muscle cells from oxidative damage (82). More importantly, these antioxidants are known to inhibit the activation of the NF-κB signaling pathway, a key regulator of pro-inflammatory cytokine production (83). Additionally, antioxidants help preserve mitochondrial integrity in neurons and myocytes, ensuring efficient energy production and preventing cell death, which is essential for maintaining the integrity of both the enteric nervous system and the pelvic floor musculature (84).

Our study has several strengths. First, we employed subgroup analyses with interaction tests to assess heterogeneity of this association across demographic and clinical strata.

Secondly, the superior discrimination capacity of GNRI on the FI in patients with stroke was observed. Thirdly, sensitivity analyses (e.g., multiple imputation, minimizing the effect of other comorbidities) confirmed the robustness of the associations. Fourth, significant mediating roles of the SII index and OBS were revealed by mediation analysis. However, this study also has several limitations. First, as a cross-sectional design, it cannot establish causal relationships between GNRI, FI, and FISI in patients with stroke. Therefore, further large-scale multicenter prospective cohort studies are required to validate the causal relationship. Second, FI-related data primarily rely on patients’ self-reported descriptions, which may introduce recall bias and potentially influence the observed associations. Third, limited data sources and a relatively homogeneous ethnic composition of the population may introduce choice bias and potentially impact the observed associations. Fourth, the mediators operate on different time scales; SII reflects acute-to-chronic inflammation, while OBS represents a stable, long-term oxidative balance. Our mediation analysis explores the mediation effect of OBS and SII, as oxidative and inflammation indicators, but cannot consider their temporal sequence. Future longitudinal studies with repeated measures are warranted. Finally, residual confounding from unmeasured factors might affect the relationships among GNRI, FI, and FISI in patients with stroke. Finally, the association between GNRI and FI could be bidirectional, mediated through biological pathways or psychosocial factors; however, cross-sectional studies cannot fully disentangle this complexity.

Future research should prioritize longitudinal studies and experimental models (e.g., animal studies) to clarify causality and explore underlying biological mechanisms.

## 5 Conclusion

Our findings demonstrate a significant inverse linear association between GNRI, FI, and FISI in patients with stroke. Furthermore, the SII index and OBS are identified as significant mediators in these relationships. Collectively, these results emphasize the critical role of anti-inflammatory and antioxidant interventions, such as increasing the intake of omega-3 fatty acids, dietary fiber, and vitamins C and E in the daily diet. Future research should prioritize designing randomized controlled trials to test the efficacy of targeted nutritional interventions in mitigating FI risk.

## Data Availability

The original contributions presented in the study are included in the article/Supplementary material, further inquiries can be directed to the corresponding author/s.
