# Supplementary material for: Association between geriatric nutritional risk index and fecal incontinence in individuals with stroke: mediating roles of systemic immune inflammation index and oxidative balance scores
Source: Front Nutr. 2025 Nov 26;12:1692314. doi: 10.3389/fnut.2025.1692314 (PMC12689289; doi:10.3389/fnut.2025.1692314)
Supplement: Supplementary file 1 [file Table_1.DOCX]

Table S1. The food list and food groups included in the 125-item food frequency questionnaire (FFQ)

| Food Group | Representative Food Items |
| --- | --- |
| Grains | Rice, steamed buns, noodles, bread, cookies, breakfast cereals, corn, sweet potatoes/potatoes, other pastries |
| Meat | Pork (lean and fatty), pork (lean), pork ribs, beef, lamb, chicken, duck, ham, sausage, bacon, other processed meats |
| Aquatic proteins | Various fish (river fish, sea fish such as hairtail, crucian carp, etc.), shrimp, crab, shellfish (clams, oysters, etc.) |
| Eggs | Eggs, duck eggs, quail eggs |
| Dairy & Bean proteins | Milk, yogurt, cheese, soy milk, tofu, dried tofu, thin tofu sheets, dried tofu sticks |
| Vegetables | Spinach, broccoli, bok choy, lettuce, water spinach, cabbage, cauliflower, broccoli, shiitake mushrooms, button mushrooms, enoki mushrooms, black fungus, carrots, daikon radishes, tomatoes, cucumbers, winter melons, pumpkins, eggplants, peppers, peas, lentils, green beans, onions, garlic, scallions, |
| Fruits | Apples, pears, bananas, citrus fruits/oranges, grapes, peaches, strawberries, watermelons, cantaloupes, mangoes, kiwis |
| Nuts | Peanuts, walnuts, almonds, melon seeds, sesame seeds |
| Beverages | Sugary beverages (such as cola, fruit drinks), tea, coffee |
| Oils & Condiments | Soybean oil, peanut oil, canola oil, olive oil, lard, salt, soy sauce, vinegar. |

**Note:** This questionnaire covers ten food categories, including grains, meat, seafood, eggs, dairy, soy products, vegetables, fruits, nuts, beverages, and cooking oils.

**Table S2.** Ingredients that make up the oxidative balance score

| **OBS components** | **Female** | | | **Male** | | | |  |
| --- | --- | --- | --- | --- | --- | --- | --- | --- |
|  | **0** | **1** | **2** | | **0** | **1** | **2** | |
| **Dietary OBS** |  |  |  | |  |  |  | |
| Dietary fiber (g/d) | < 9.95 | 9.95-15.50 | ≥ 15.50 | | < 11.4 | 11.40-17.75 | ≥ 17.75 | |
| Carotene (RE/d) | < 454.83 | 454.83-1602.83 | ≥ 1602.83 | | < 417 | 417.00-1441.50 | ≥ 1441.50 | |
| Vitamin B6 (mg/d) | < 1.15 | 1.15-1.71 | ≥ 1.71 | | < 1.43 | 1.43-2.16 | ≥ 2.16 | |
| Vitamin B12 (mcg/d) | < 2.30 | 2.30-3.94 | ≥ 3.94 | | < 3.05 | 3.05-5.24 | ≥ 5.24 | |
| Riboflavin (mg/d) | < 1.22 | 1.22-1.74 | ≥ 1.74 | | < 1.46 | 1.46-2.15 | ≥ 2.15 | |
| Total Folate (mcg/d) | < 224.00 | 224.00-337.16 | ≥ 337.16 | | < 270.00 | 270.00-409.50 | ≥ 409.50 | |
| Niacin (mg/d) | < 14.92 | 14.92-21.53 | ≥ 21.53 | | < 18.81 | 18.81-27.97 | ≥ 27.97 | |
| Vitamin C (mg/d) | < 37.60 | 37.60-83.06 | ≥ 78.50 | | < 28.20 | 38.20-89.65 | ≥ 89.65 | |
| Vitamin E (ATE) (mg/d) | < 5.33 | 5.33-8.21 | ≥ 8.21 | | < 5.98 | 5.98-9.43 | ≥ 9.43 | |
| Calcium (mg/d) | < 609.00 | 609.00-906.00 | ≥ 906.00 | | < 720.00 | 720.00-1090.00 | ≥ 1090.00 | |
| Magnesium (mg/d) | < 185.00 | 185.00-261.00 | ≥ 261.00 | | < 214.00 | 214.00-311.50 | ≥ 311.50 | |
| Zinc (mg/d) | < 6.34. | 6.34.-9.20 | ≥ 9.20 | | < 7.96 | 7.96-11.87 | ≥ 11.87 | |
| Copper (mg/d) | < 0.70 | 0.70-1.01 | ≥ 1.01 | | < 0.78 | 0.78-1.19 | ≥ 1.19 | |
| Selenium (mcg/d) | < 68.35 | 68.35-98.23 | ≥ 98.23 | | < 84.80 | 84.80-126.40 | ≥ 126.40 | |
| Iron (mg/d) | ≥ 12.82 | 8.83-12.82 | < 8.83 | | ≥ 10.77 | 10.77-15.96 | < 15.96 | |
| Total fat (gm/d) | ≥ 78.38 | 53.83-78.38 | < 53.83 | | ≥ 97.04 | 64.18-97.04 | < 64.18 | |
| **Lifestyle OBS** |  |  |  | |  |  |  | |
| Physical activity  (MET-minutes/week) | < 225.00 | 225.00-720.00 | ≥720.00 | | < 360.00 | 360.00-1140.00 | ≥ 1140.00 | |
| Cotinine (ng/mL) | ≥ 0.09 | 0.01-0.09 | < 0.01 | | ≥ 0.40 | 0.02-0.40 | < 0.02 | |
| Alcohol (g/d) | ≥ 15.00 | (0, 15.00) | non | | ≥ 30.00 | (0, 30.00) | non | |
| Body mass index(kg/m2) | ≥ 30.60 | [23.00, 30.60) | < 23.00 | | ≥ 29.10 | [22.80, 29.10) | < 22.80 | |

**Abbreviations:** RE: retinal equivalent; ATE: alpha-tocopherol equivalent; MET: metabolic equivalent. OBS: oxidative balance score. Note: For each component, intake levels or status were categorized into tertiles, quartiles, or based on clinical guidelines. Antioxidant components were scored from 0 to 2, where higher intakes received higher scores. Conversely, pro-oxidant components were scored from 2 to 0, where higher intakes received lower scores. The scores across all 20 components were summed to yield the overall OBS.

|  | Two or more  times per day | Once  per Day | Two or more  times per week | Once per week | One to three  times per month | Never |
| --- | --- | --- | --- | --- | --- | --- |
| Gas Stool | 9 | 8 | 6 | 4 | 2 | 0 |
| Mucus Stool | 11 | 9 | 7 | 7 | 5 | 0 |
| Liquid Stool | 18 | 16 | 14 | 13 | 10 | 0 |
| Solid Stool | 19 | 17 | 16 | 14 | 11 | 0 |

**Table S3.** The weight scores of four incontinences in our cross-sectional study

Note: The severity of incontinence was assessed using a weighted scoring system across four primary subtypes: gas stool, mucus stool, liquid stool, and solid stool. For each subtype, a weight score was assigned based on the frequency of leakage episodes. The total incontinence burden for an individual could be represented by the separate scores for each subtype or by a composite sum score.

**Table S4.** Association of GNRI with FISI in individuals with stroke stratified by various subgroups

| Subgroups | β (95%CI) | *P*-value | *P* for interaction |
| --- | --- | --- | --- |
| Gender  Female  Male | -1.44 (-2.47, -0.43)  -1.09 (-2.07, -0.21) | < 0.001  < 0.001 | 0.437 |
| Educational levels  Less than High-school  High school  College or above | -1.43 (-2.09, -0.79)  -1.36 (-2.25, -0.76)  -1.49 (-2.28, -0.56) | < 0.001  < 0.001  < 0.001 | 0.378 |
| Poverty index ratio  PIR < 1  1 ≤ PIR < 3  PIR ≥ 3 | -1.51 (-2.65, -0.74)  -0.26 (-0.78, 0.43)  -1.42 (-2.46, -0.53) | < 0.001  0.323  < 0.001 | 0.003 |
| BMI  Underweight  Normal weight  Overweight  Obesity | -1.21 (-2.13, -0.12)  -0.24 (-0.67, 0.58)  -1.32 (-2.41, -0.42)  -1.27 (-2.45, -0.48) | 0.001  0.387  < 0.001  < 0.001 | 0.002 |
| Drinking status  Yes  No | -0.56 (-1.03, 0.13)  -0.48 (-1.22, 0.27) | 0.213  0.435 | 0.782 |
| Smoking status  Never  Former  Current | -0.26 (-0.75, 0.48)  -1.06 (-1.96, -0.21)  -1.18 (-2.27, -0.28) | 0.265  < 0.001  < 0.001 | 0.007 |
| Physical levels  Vigorous  Middle  Other | -1.18 (-2.01, -0.45)  -1.06 (-2.13, -0.25)  -1.15 (-2.28, -0.36) | < 0.001  < 0.001  < 0.001 | 0.652 |
| Hypertension  Yes  No | -1.02 (-2.17, -0.31)  -0.89 (-1.81, -0.12) | < 0.001  0.001 | 0.427 |
| Diabetes  Yes  No | -1.19 (-2.31, -0.08)  -0.37 (-0.86, 0.22) | 0.015  0.278 | 0.004 |
| COPD  Yes  No | -0.69 (-1.51, 0.18)  -1.25 (-2.26, -0.19) | 0.135  < 0.001 | 0.089 |
| CKD  Yes  No | -0.79 (-1.67, -0.18)  -0.85 (-2.16, -0.22) | < 0.001  <0.001 | 0.617 |

**Abbreviations:** BMI: body mass index, COPD: chronic obstructive pulmonary disease, CKD: Chronic Kidney Disease, PIR: Poverty index ratio. Notes: All covariates were adjusted in the model.

**Table S5.** Association of the SII index and OBS with FI and FISI in individuals with stroke

|  | OR (95%CI) | *P*-value | β (95%CI) | *P*-value |
| --- | --- | --- | --- | --- |
| SII index |  |  |  |  |
| Continuous | 1.45 (1.13, 1.89) | 0.002 | 0.72 (0.34, 1.36) | < 0.001 |
| Q1 | *Ref* (1) |  | *Ref* (0) |  |
| Q2 | 1.08 (0.91, 1.36) | 0.324 | 0.17 (-0.24, 0.65) | 0.476 |
| Q3 | 1.27 (1.06, 1.62) | 0.011 | 0.45 (0.12, 0.94) | 0.001 |
| Q4 | 1.64 (1.21, 2.01) | < 0.001 | 0.98 (0.37, 1.69) | < 0.001 |
| *P* for trend | 0.003 |  | 0.001 |  |
| OBS |  |  |  |  |
| Continuous | 0.73 (0.54, 0.91) | < 0.001 | -0.58 (-1.48, -0.21) | < 0.001 |
| Q1 | *Ref* (1) |  | *Ref* (0) |  |
| Q2 | 0.92 (0.78, 1.12) | 0.576 | -0.18 (-0.78, 0.29) | 0.398 |
| Q3 | 0.76 (0.50, 0.95) | 0.002 | -0.99 (-1.78, -0.16) | 0.001 |
| Q4 | 0.59 (0.37, 0.81) | < 0.001 | -1.54 ( -2.32, -0.66) | < 0.001 |
| *P* for trend | 0.001 |  | < 0.001 |  |
| SII × OBS | 1.24 (1.06, 1.59) | 0.001 | 0.16 (0.02, 0.45) | 0.003 |

**Abbreviations:** SII: systemic immune-inflammation index, OBS: oxidative balance score. Q1: quartile 1; Q2: quartile 2; Q3: quartile 3; Q4: quartile 4. **Notes**: *Ref:* reference; all covariates were adjusted in the model. SII × OBS was regarded as the interaction effect of SII and OBS. *P* <0.05 was regarded as having statistical significance.


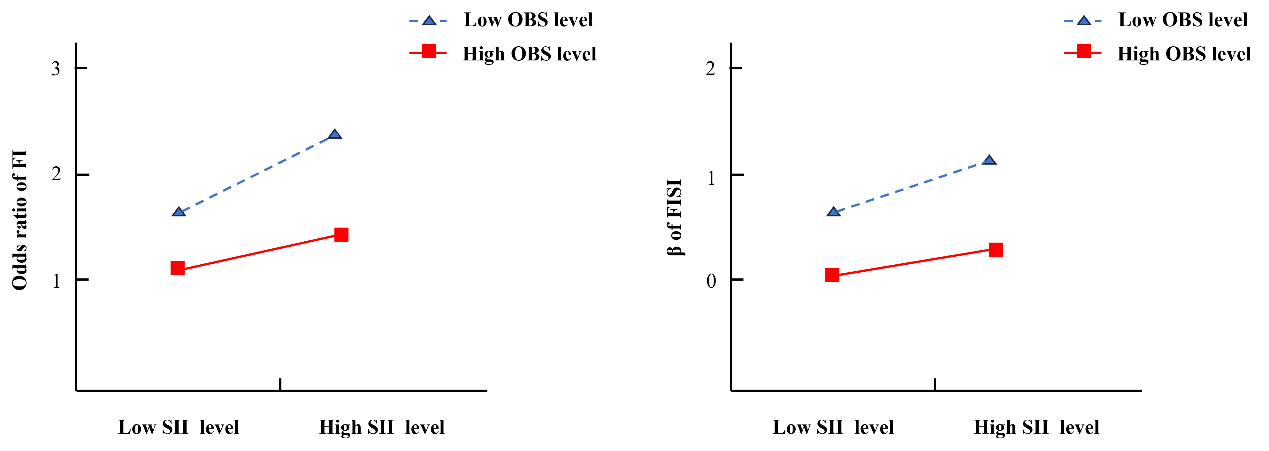


**Fig S1** Interaction effect of SII and OBS on FI and FISI. Simple slopes analysis showed that OR of FI and β of FISI increased more sharply with SII among individuals with low OBS level (blue dashed line), whereas the decline is smaller among those with high OBS level (red solid line).

|  | β (95%CI) | *P*-value |
| --- | --- | --- |
| SII index |  |  |
| Continuous | -0.56 (-0.98, -0.23) | < 0.001 |
| Q1 | *Ref* (0) |  |
| Q2 | -0.24 (-0.56, -0.01) | 0.041 |
| Q3 | -0.62 (-1.21, -0.12) | 0.001 |
| Q4 | -0.89 (-1.63, -0.31) | < 0.001 |
| *P* for trend | 0.001 |  |
| OBS |  |  |
| Continuous | 0.78 (0.23, 1.64) | < 0.001 |
| Q1 | *Ref* (0) |  |
| Q2 | 0.26 (0.11, 0.46) | 0.001 |
| Q3 | 0.54 (0.32, 0.87) | < 0.001 |
| Q4 | 1.02 (0.58, 1.79) | < 0.001 |
| *P* for trend | < 0.001 |  |
| SII × OBS | 0.20 (0.03,0.45) | <0.001 |

**Table S6.** Association of the SII index and OBS with GNRI in individuals with stroke

**Abbreviations:** SII: systemic immune-inflammation index, OBS: oxidative balance score. Q1: quartile 1; Q2: quartile 2; Q3: quartile 3; Q4: quartile 4. Notes: *Ref:* reference; all covariates were adjusted in the model. *P* <0.05 was regarded as having statistical significance.


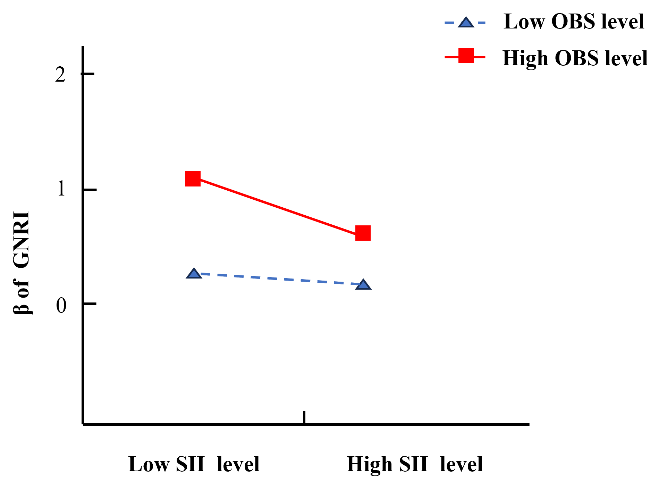


**Fig S2** Interaction effect of SII and OBS on GNRI. Simple slopes analysis shows that β of GNRI decreases more sharply with SII among individuals with high OBS level (red solid line), whereas the decline is smaller among those with low OBS level (blue dashed line).

**Table S7.** Association of GNRI with FI and FISI after using multiple interpolation methods to fill in the missing data

|  | OR (95%CI) | *P*-value | β (95%CI) | *P*-value |
| --- | --- | --- | --- | --- |
| Model Ⅰ |  |  |  |  |
| Continuous | 0.63 (0.51, 0.79) | < 0.001 | -1.21 (-1.78, -0.76) | < 0.001 |
| Q1 | *Ref* (1) |  | *Ref* (0) |  |
| Q2 | 0.87 (0.72, 1.05) | 0.323 | -0.47 (-0.99, 0.16) | 0.642 |
| Q3 | 0.72 (0.61, 0.90) | 0.008 | -0.99 (-1.26, -0.18) | 0.002 |
| Q4 | 0.46 (0.31, 0.59) | < 0.001 | -1.82 (-2.65, -1.45) | < 0.001 |
| *P* for trend | < 0.001 |  | < 0.001 |  |
| Model Ⅱ |  |  |  |  |
| Continuous | 0.68 (0.52, 0.84) | < 0.001 | -1.31 (-1.87, -0.52) | < 0.001 |
| Q1 | *Ref* (1) |  | *Ref* (0) |  |
| Q2 | 0.86 (0.70, 1.03) | 0.279 | -0.44 (-1.04, 0.12) | 0.451 |
| Q3 | 0.75 (0.61, 0.86) | 0.005 | -0.81 (-1.33, -0.44) | < 0.001 |
| Q4 | 0.52 (0.41, 0.67) | < 0.001 | -1.73 (-2.40, -1.21) | < 0.001 |
| *P* for trend | < 0.001 |  | <0.001 |  |
| Model Ⅲ |  |  |  |  |
| Continuous | 0.73 (0.59, 0.87) | < 0.001 | -1.12 (-1.67, -0.43) | < 0.001 |
| Q1 | *Ref* (1) |  | *Ref* (0) |  |
| Q2 | 0.92 (0.78, 1.11) | 0.512 | -0.26 (-1.01, 0.33) | 0.349 |
| Q3 | 0.80 (0.62, 0.95) | 0.012 | -0.65 (-1.27, -0.10) | < 0.001 |
| Q4 | 0.61 (0.48, 0.72) | < 0.001 | -1.25 (-2.13, -0.52) | < 0.001 |
| *P* for trend | < 0.001 |  | <0.001 |  |

**Abbreviations:** Q1: quartile 1; Q2: quartile 2; Q3: quartile 3; Q4: quartile 4. Notes: *Ref*: reference; Model Ⅰ is a crude model. Model II adjusted for age, gender, educational levels, and PIR. Model Ⅲ adjusted for all covariates. *P* <0.05 was regarded as having statistical significance.

**Table S8**. Association of GNRI with FI and FISI after deleting those with hypertension and diabetes, COPD, and CKD.

|  | OR (95%CI) | *P*-value | β (95%CI) | *P*-value |
| --- | --- | --- | --- | --- |
| Model Ⅰ |  |  |  |  |
| Continuous | 0.68 (0.54, 0.81) | < 0.001 | -1.16 (-1.70, -0.62) | < 0.001 |
| Q1 | *Ref* (1) |  | *Ref* (0) |  |
| Q2 | 0.90 (0.75, 1.08) | 0.381 | -0.29 (-0.72, 0.19) | 0.441 |
| Q3 | 0.78 (0.64, 0.92) | 0.016 | -0.70 (-1.21, -0.12) | 0.004 |
| Q4 | 0.53 (0.36, 0.65) | <0.001 | -1.61 (-2.43, -1.31) | < 0.001 |
| *P* for trend | < 0.001 |  | <0.001 |  |
| Model Ⅱ |  |  |  |  |
| Continuous | 0.72 (0.58, 0.85) | 0.001 | -1.09 (-1.48, -0.56) | < 0.001 |
| Q1 | *Ref* (1) |  | *Ref* (0) |  |
| Q2 | 0.92 (0.77, 1.11) | 0.429 | -0.25 (-0.69, 0.22) | 0.509 |
| Q3 | 0.80 (0.66, 0.93) | 0.009 | -0.67 (-1.14, -0.10) | 0.008 |
| Q4 | 0.56 (0.45, 0.70) | < 0.001 | -1.41 (-1.89, -0.86) | < 0.001 |
| *P* for trend | < 0.001 |  | <0.001 |  |
| Model Ⅲ |  |  |  |  |
| Continuous | 0.76 (0.63, 0.89) | 0.005 | -0.89 ( -1.21, -0.48) | < 0.001 |
| Q1 | *Ref* (1) |  | *Ref* (0) |  |
| Q2 | 0.95 (0.81, 1.15) | 0.602 | -0.16 (-0.58, 0.28) | 0.349 |
| Q3 | 0.84 (0.70, 0.96) | 0.035 | -0.54 (-1.01, -0.06) | 0.012 |
| Q4 | 0.65 (0.52, 0.77) | < 0.001 | -1.17 (-1.69, -0.67) | < 0.001 |
| *P* for trend | < 0.001 |  | <0.001 |  |

**Abbreviations:** Q1: quartile 1; Q2: quartile 2; Q3: quartile 3; Q4: quartile 4. Notes: *Ref*: reference; Model Ⅰ is a crude model. Model II adjusted for age, gender, educational levels, and PIR. Model Ⅲ adjusted for all covariates. *P* <0.05 was regarded as having statistical significance.
